# Subcutaneous emphysema during mandibular wisdom tooth extraction: Cases series

**DOI:** 10.1016/j.amsu.2021.103039

**Published:** 2021-11-11

**Authors:** D. Sarfi, S. Haitami, M. Farouk, I. Ben Yahya

**Affiliations:** Oral Surgery, Dental Consultation and Treatment Center, Ibn Rochd University Hospital Center, Casablanca, BP, 9157, Morocco

**Keywords:** Subcutaneous emphysema, Dental procedure, Dysphagia, Dental emergency

## Abstract

Subcutaneous emphysema occurs when air is forced under the tissue, causing swelling, crepitus on palpation, and the possibility of spreading along the fascial planes.

Although subcutaneous emphysema secondary to dental procedures is rare, it can be a potentially fatal complication if not diagnosed and treated promptly and correctly.

Dentists need to be able to differentiate subcutaneous emphysemas from more common disease processes that have similar clinical presentations.

We report a 22-year-old male who underwent mandibular wisdom tooth extraction and subsequently developed extensive subcutaneous emphysema. The patient was quickly taken care of, in partnership with the maxillofacial department. The purpose of this report is to bring attention to the fact that obtaining an accurate diagnosis for this condition is very important and management on time can prevent serious complications.

## Highlights

• Describe subcutaneous emphysema as a medical emergency.
• To highlight the pathognomonic signs of subcutaneous emphysema.
• The importance of rapid management of subcutaneous emphysema.

## 1 Introduction

Subcutaneous emphysema is a rare but serious side effect of dental and oral surgery procedures [1].

The condition is characterized by air being forced underneath the tissue, leading to crepitus on palpation, swelling, and with potential to spread along the fascial planes to the periorbital, mediastinal, pericardial, and/or thoracic spaces. A wide range of causes has been documented for the origin of subcutaneous emphysema during dental treatment including crown preparations, endodontic therapy, extractions, as well as oral surgery procedures [1].

The use of air-driven handpieces appears to be related in the majority of the case reports; High-speed air turbine drills are used to section the tooth to facilitate extraction and are driven by compressed air at 3.5–4.0 kg/cm2, rotating at 450,000 rpm [2].

However, events in the perioperative period including endotracheal intubation and positive pressure ventilation have also been reported in association with subcutaneous emphysema [1].

The following report presents a case of subcutaneous emphysema that occurred during third molar extraction with the use of an air turbine handpiece, in the Department of Oral Surgery, Dental Consultation and Treatment Center, Ibn Rochd University Hospital Center, Casablanca, Morocco. Case management is described and issues relative to the diagnosis and prevention of this surgical complication are discussed.

## 2 Methods

The research registry number in accordance with the Declaration of Helsinki is researchregistry7204 (https://www.researchregistry.com/browse-the-registry).

The prospective study described in our article is a case series, and is unique.

All our patients were treated in a hospital setting, by Dr Sarfi and Pr Haitami (Professor, D.M.D., Specialist in Oral Medicine and Oral Surgery), at the Center for Dental Consultations and Treatments at the CHU Ibn Rochd Hospital in Casablanca.

All of our patients were recruited between 2019 and 2021, and received a follow-up that lasted up to 1 month.

No particular precautions were taken before the intervention.

**‘This case series has been reported in line with the PROCESS Guideline** [16]

## 3 Case report 1

**Fig. 1:**
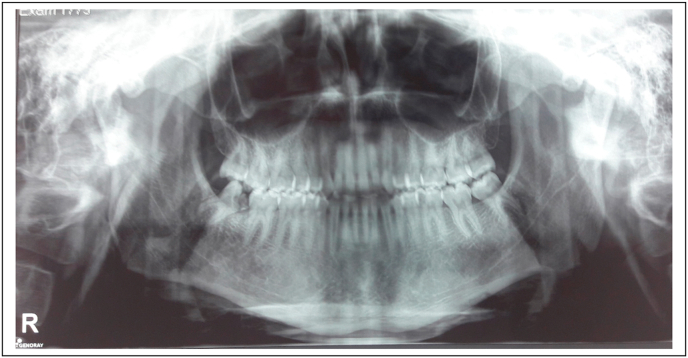
Preoperative radiograph showing the impacted mandibular right third molar.

A 22-year-old male patient was referred to our Department by her private dentist for extraction of the mandibular right third molar, with a mesial carious lesion, which was impacted in the mandible, in an oblique position, with a curved distal root and a straight mesial root (Fig. 1). Medical history was not relevant for systemic conditions. He denied tobacco or alcohol use Oral and physical examinations, including panoramic radiography, were performed. Based on these findings, surgery was scheduled under local anesthesia.

Using a Bard-Parker #15 scalpel blade, the surgical flap was made with a release incision mesially to the second molar and adjacent to the papilla. To expose the overlying bone, the mucoperiosteal flap was lifted and held with a surgical retractor. With copious irrigation, osteotomy was performed using a Lindemann stainless steel bur on an air-driven high-speed turbine.

**Fig. 2:**
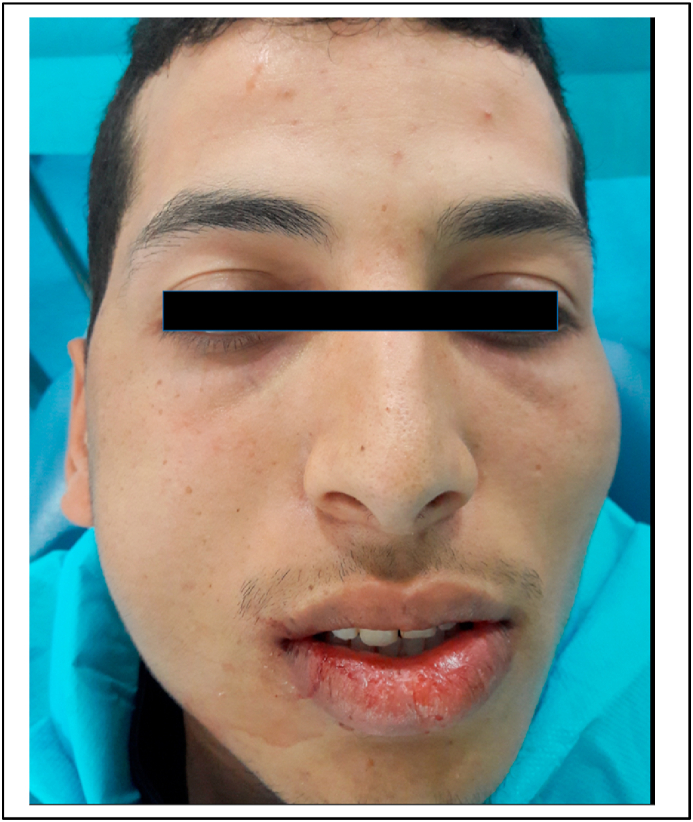
Clinical features of subcutaneous emphysema immediately after its occurrence.

**Fig. 3:**
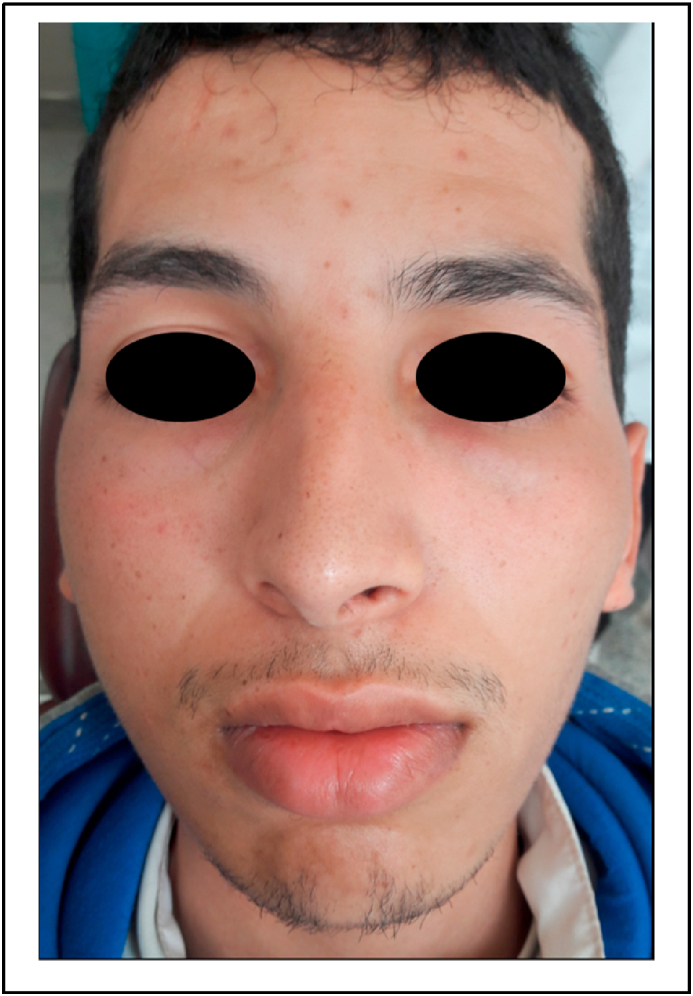
Considerable reduction of the swelling 2 days after the procedure.

**Fig. 4:**
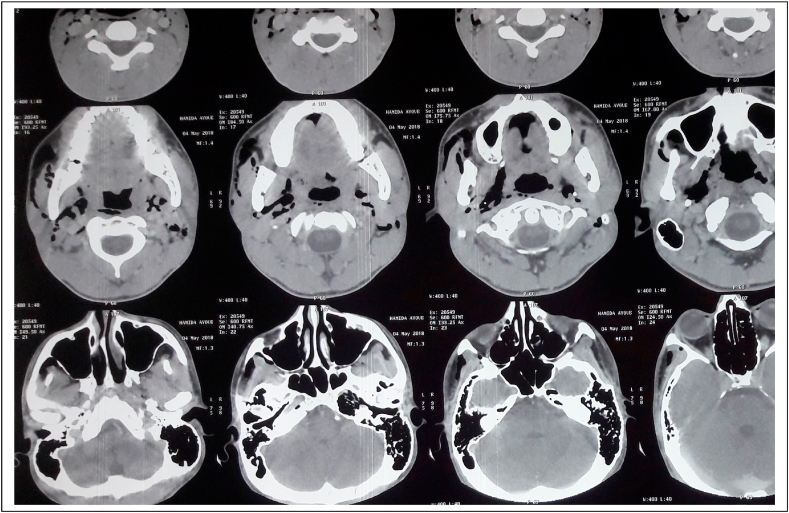
Cervicofacial CT showing emphysema of the deep facial and soft tissue spaces.

**Fig. 5:**
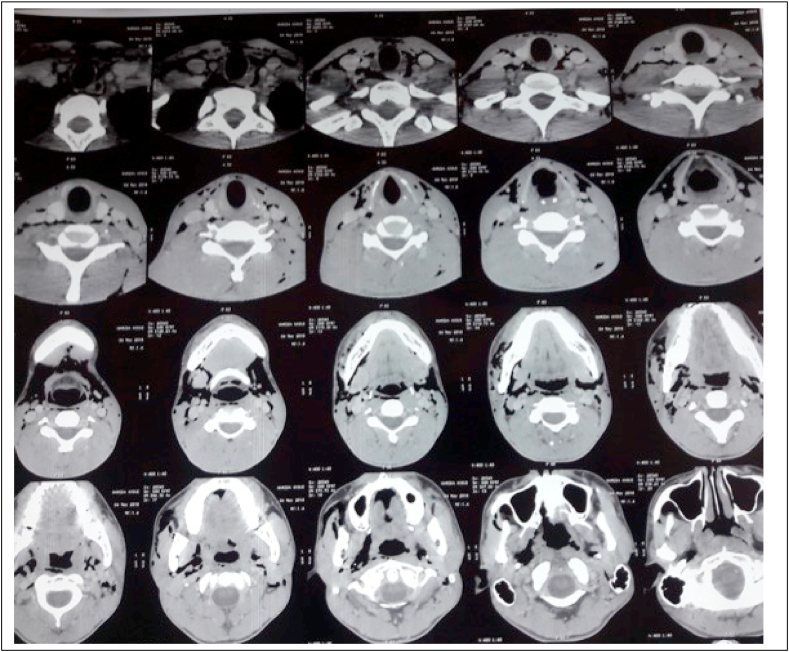
Cervicofacial CT showing emphysema of the deep facial and soft tissue spaces.

During this surgical phase, the air turbine handpiece was inclined towards the buccal face of the tooth. The right side of the patient's face became swollen. (Fig. 2). The area was non-erythematous and non-tender. On palpation, there was a sensation of crepitation of the swelling, which had slightly closed the patient's eye. The patient did not have a lot of pain, tenderness to palpation, or difficulty breathing as a result of the swelling. The subcutaneous emphysema was located in the left lateral neck, cheek, and orbital regions. The surgical intervention was immediately suspended, and the flap was sutured using a 3/0 silk suture. The patient was kept under observation for 1 h and prednisolone 60 mg per day for 5 days, antibiotic (amoxicillin 3 gr/day for 7 days) and analgesic (paracetamol 1g every 6 h for 3 days) therapy was prescribed. The patient returned to the clinic 2 days later, complaining of discomfort on swallowing and speaking. The swelling had considerably reduced in volume. (Fig. 3). The patient was referred directly to the maxillofacial department. The patient's blood pressure; heart rate; respiratory rate; temperature, and oxygen saturation were normal. Cervicofacial CT showed emphysema of the deep spaces of the face in the retropharyngeal space, the lateral part of the pharyngeal space, and the right infratemporal fossa. There is associated soft tissue emphysema in the right jugal and submandibular region and the upper mediastinal region. We also noted a discrete thickening of the jugal and submandibular soft tissues on the right without any detectable collection(Fig. 4, Fig. 5). The treatment plan was a close observation of the airway, in the maxillofacial department, under the same drug prescription. Complete remission was observed about 5 days after. Prednisolone 60 mg per day for 5 days, antibiotic (amoxicillin 3 gr/day for 7 days), and analgesic (paracetamol 1g every 6 h for 3 days) therapy were prescribed. After 3 days, complete remission was noted in the patient.

## 4 Case report 2

**Fig. 6:**
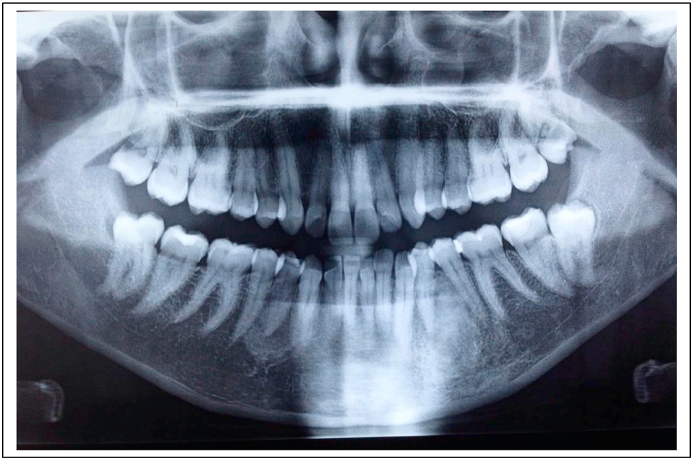
Preoperative radiograph showing the impacted mandibular left third molar.

A 24-year-old woman, with no medical history, consulted for the extraction of an enclosed 38 following episodes of repetitive pericoronitis and difficulty chewing. Oral and physical examinations, including panoramic radiography (Fig. 6), were performed. Surgery was scheduled under local anesthesia.

**Fig. 7:**
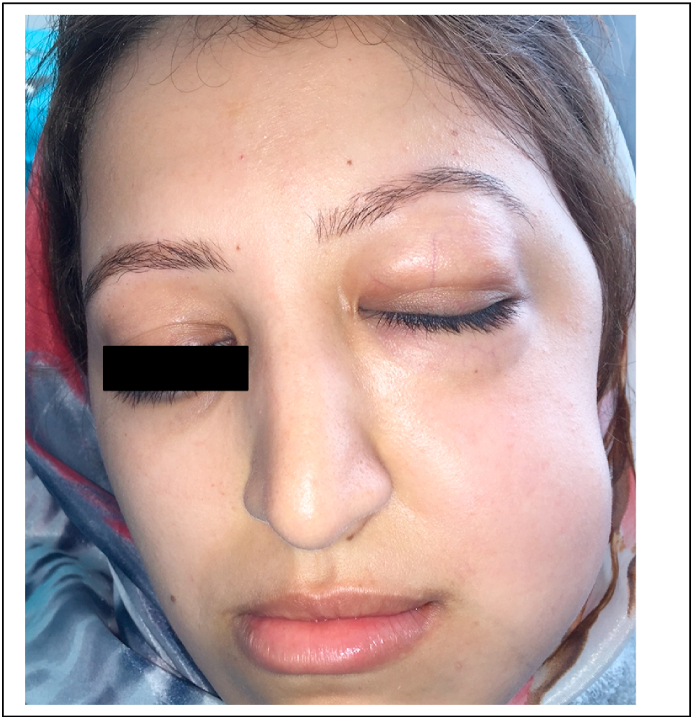
Exobuccal examination showing swelling of the patient's left jugal surface, with closure of her eye.

After detachment of the flap, and during the osteotomy, a swelling suddenly appeared on the whole of the patient's left jugal face, which resulted in the total closure of her left eye. Like the previous case, the swelling was neither painful nor erythematous. The swelling immediately closed the patient's right eye. However, crepitations were noted during palpation (Fig. 7). The diagnosis of emphysema has been made. The surgery was immediately interrupted, so the flap was sutured, and prednisolone 60 mg per day for 5 days, antibiotics (amoxicillin 3 gr/day for 7 days), and analgesic (paracetamol 1g every 6 h for 3 days) therapy were prescribed. Complete remission was noted 5 days later, with a complete disappearance of the tumor, and without any complications.

## 5 Discussion

The first report of subcutaneous emphysema, was published by Turnbull in 1900, related to a dental procedure (a third molar extraction) [3].

Dental procedures that cause an interruption in the mucosa of the oral cavity and introduce air into the connective tissue spaces of the head and neck can lead to subcutaneous emphysema. There is an increased risk for the spreading of bacteria and the development of life-threatening infections of the retropharyngeal space and the mediastinum, due to the communication of the facial spaces with the mediastinum [4].

Although this is mostly benign, there is a risk of progression to more serious consequences including pneumothorax, air embolism, mediastinitis, cranial nerves palsy, and cardiac tamponade [5,1].

Water and air may then dissect along the multiple fascial planes between the mouth and mediastinum, especially near the roots of the 3 M that directly communicate with the sublingual and retropharyngeal spaces, risking the spread of contaminants from the gingival flora into the mediastinum. A mediastinal extension is associated with several potentially serious complications that increase morbidity and mortality, such as infectious mediastinitis, tension pneumothorax, pericardial tamponade, airway obstruction, and even air embolism [6] Surgery is not the only procedure at risk for the development of subcutaneous emphysema, as cases have been described during restorative procedures, crown preparation, and endodontic treatment. Emphysema has also been reported during oral laser surgery procedures.

Elevation of a large flap can raise the risk of provoking emphysema, especially during third molar surgery [2] Subcutaneous emphysema can also be induced by the patient coughing, blowing forcefully, smoking, or vomiting after a dental procedure [1].

The pressure that increases air diffusion can be raised by simple actions: blowing the nose, sneezing … [3]. In this case, the air could have been forced into the subcutaneous tissue during the sectioning of the tooth with the high-speed air turbine handpiece and the elevated valve could have caused a valve effect as described in other clinical situations.

Signs and symptoms of subcutaneous emphysema after surgery vary. While some reports show almost immediate signs of swelling, in other cases it takes a few hours after the end of the procedure for the patient to become symptomatic (90% of cases). Very rarely, these symptoms do not appear until after 48 h [9].. In our case, the swelling had appeared during the procedure, but the discomfort to swallow and speak appeared only after 2 days.

Physicians might attribute immediate dyspnea and swelling after a dental procedure to allergic reaction or angioedema, and delayed symptoms to hematoma or soft tissue infection such as cellulitis, Ludwig's angina or Lemierre's syndrome. Patients with isolated subcutaneous emphysema typically present with painless edema of the face and neck; however, the presence of palpable crepitus is pathognomonic and clearly distinguishes from other causes [17].

Patients with subcutaneous emphysema have marked swelling and discomfort where the air has entered the subcutaneous tissue.

They may also have breathing difficulties if the subcutaneous emphysema has spread to the paratracheal, mediastinal, or thoracic spaces.

The pathognomonic sign of subcutaneous emphysema is crepitus on palpation [[1], [7]] [][[1], [7][]]. Once palpable crepitus is noted, there should be immediate consideration for pneumomediastinum and potentially associated pneumothorax, esophageal rupture, or infection within the fascial planes. Patients with pneumomediastinum typically present with dyspnea, chest pain, back pain, dysphagia, odynophagia, or Hamman's sign (a systolic friction rub) [17].

Rarely, dyslalia, dysphonia, brassy voice, and hearing loss occur due to free air in the retropharyngeal space compressing the Eustachian tube [10].

A CT of the chest and neck is the most sensitive test for detecting widespread emphysema and pneumomediastinum [6].

Subcutaneous emphysema, with most cases spontaneously resolving within 2–10 days. Like our patient who showed complete remission after a few days of the procedure. The rationale for antibiotics is that air introduced from an intraoral site is likely to carry with it bacteria that could potentially lead to rapidly spreading cellulitis or necrotizing fasciitis [1].

Patients with pneumomediastinum, however, are typically admitted for IV prophylactic, broad-spectrum antibiotics to cover oral aerobes and anaerobes.

As with antibiotic therapy, it is difficult to determine whether the use of steroids has any benefit in the treatment of subcutaneous emphysema. Consensus on antibiotic therapy and corticosteroid administration after subcutaneous emphysema is unclear [1].

The possibility of further complications may be decreased by use of sedatives to lower respiratory effort. Additionally, stool softeners, antitussives, and antihistamines to decrease intrathoracic pressure generated from Valsalva, coughing, and nose blowing may be efficacious [17].

In most cases, the reabsorption of air begins within two to three days, frequently having complete resolution by day 7–10 after onset [8]. This process may be hastened with the use of oxygen inhalation through the nasal cannula, which reduces the partial pressure of nitrogen within the blood, ultimately increasing air reabsorption [10].

Subcutaneous emphysema can sometimes lead to significant discomfort, disfigurement, and anxiety. In these situations, micro-drainage could be considered. This is a simple, minimally invasive procedure accomplished by placing a subcutaneous fenestrated catheter into the area of subcutaneous emphysema and performing sequential massage. It is reported to improve symptoms within hours and has a low chance of complications [11].

Prevention of emphysema requires adherence to well-accepted surgical procedures. mucoperiosteal flap elevation should be minimal and should not extend to the lingual alveolus of the mandibular third molar area. muscle attachments should be preserved whenever possible. Firm but gentle retraction of mucoperiosteal flaps protects soft tissues from cutting instruments. **Further research is needed to better prevent this incident, as well as to improve its management (item 7b)**

Moreover, air turbine high-speed handpieces should not be used for longer than required [15]. It is preferable to use a contra-angle multiplier (1:5) handpiece, which does not use flux of high-speed air for movement. In every case, prompt diagnosis is important to avoid life-threatening consequences such as pneumomediastinum or pneumothorax [14].

## 6 Conclusion

Although rare, iatrogenic subcutaneous emphysema can have serious and potentially life-threatening effects. Therefore, caution should be exercised when using compressed air handpieces. When subcutaneous emphysema does arise, it must be quickly diagnosed and properly managed to reduce further complications.

Clinicians should maintain proper maintenance of the pneumatic turbine to prevent subcutaneous emphysema. Additionally, postoperative instructions after a dental or surgical procedure should include avoidance of any activity that may increase pressure in the oral cavity like coughing, smoking, blowing the nose, using straws, or vomiting.

## Ethical approval

Written informed consent was obtained from the patient for publication of this case report and accompanying images. A copy of the written consent is available for review by the Editor-in-Chief of this journal on request.

## Sources of funding

The authors declared that this study has received no financial support.

## Consent

Written informed consent was obtained from the patient for publication of this case report and accompanying images. A copy of the written consent is available for review by the Editor-in-Chief of this journal on request.

## Registration of research studies

1. Name of the registry: Researchregistry7204
2. Unique Identifying number or registration ID: 7204
3. Hyperlink to your specific registration (must be publicly accessible and will be checked):

## Guarantor

DOUNIA SARFI.

## Declaration of competing interest

Authors of this article have no conflict or competing interests. All of the authors approved the final version of the manuscript.

## Declaration of competing interest

Authors of this article have no conflict or competing interests. All of the authors approved the final version of the manuscript.
