# Supplementary material for: Subcutaneous emphysema during mandibular wisdom tooth extraction: Cases series
Source: Ann Med Surg (Lond). 2021 Nov 11;72:103039. doi: 10.1016/j.amsu.2021.103039 (PMC8591463; doi:10.1016/j.amsu.2021.103039)
Supplement: Multimedia component 1 [file mmc1.docx]

| **PROCESS 2020 Checklist** | | | |
| --- | --- | --- | --- |
| **Topic** | **Item** | **Checklist Item Description** | **Page Number** |
| **Title** | **1** | - The phrase ‘case series’ and the area of focus should appear in the title (e.g. patient population, diagnosis, intervention or outcome). | 1 |
| **Key Words** | **2** | - Include three to six keywords that identify what is covered in the case series (e.g. patient population, diagnosis, intervention or outcome). - Include ‘case series’ as one of the keywords. | 1 |
| **Abstract** | **3a** | Introduction and Importance   - Describe what is unique or educational. - What is the overarching theme of the case series? | 1 |
|  | **3b** | Methods   - Describe what was done, how and when was it done and by whom. | 1 |
|  | **3c** | Outcomes   - Describe the outcomes of the intervention and management strategy. | 1 |
|  | **3d** | Conclusion   - Describe the take home message(s), including what has been learnt? - How will this impact future clinical practice? | 1 |
| **Introduction** | **4** | - Describe the background of the case series and specify the overarching theme (e.g. common disease, intervention, or outcome). - The introduction should explain what is unique or educational about the case series. - Relevant scientific literature should be referenced. - Introduction should be 1-2 paragraphs in length. | 1,2 |
| **Methods** | **5a** | Registration   - State the research registry number in accordance with the Declaration of Helsinki - "Every research study involving human subjects must be registered in a publicly accessible database". This can be obtained from, for example, ResearchRegistry.com, ClinicalTrials.gov, or ISRCTN. - If a protocol already exists, state the corresponding registration number and access directions (e.g. website or journal, and include a hyperlink that is publicly accessible). It must be written in the English language. | 2 |
|  | **5b** | Study Design   - State that the study is a case series. - State whether the case series is: (1) prospective/ retrospective, (2) single/multi-centre, and if (3) cases are consecutive/non-consecutive. | 2 |
|  | **5c** | Settings and Time-Frames   - Describe the setting(s) in which the patient was managed (e.g. research institution, teaching/district general hospital, community, or private practice). - Document any relevant dates (e.g. recruitment, intervention, follow-up, and data collection time-frames). | 2 |
|  | **5d** | Participants   - Describe the relevant characteristics (e.g. demographics, comorbidities, tumour staging, smoking status) and if relevant, exposure(s) of the participants. - Describe the method of participant recruitment, if relevant. - State any subsequent inclusion or exclusion criteria, and how the participants were selected. - Methods used to ensure the de-identification of patient information. | 2 |
|  | **5e** | Pre-Intervention Patient Optimisation   - Lifestyle (e.g. weight loss). - Medication review (e.g. anticoagulation, oral hypoglycemics/insulin). - Pre-surgical stabilisation/preparation (e.g. treating hypothermia/hypovolemia/hypotension, ICU care for sepsis, nil by mouth, or enema). - Other (e.g. psychological support). | 2 |
|  | **5f** | Interventions   - Describe the type(s) of intervention(s) used (e.g. pharmacological, surgical, physiotherapy, psychological, preventative). - Describe any concurrent treatments (e.g. antibiotics, analgesia, antiemetics, venous thromboembolism prophylaxis). | 2,3,4 |
|  | **5g** | Intervention Details   - Describe the rationale behind the treatment offered, how it was performed and time to intervention. - For pharmacological therapies, include information on the formulation, dosage, strength, route, and duration. - For surgery, include details such as anaesthesia, patient position, preparation used, use of other relevant equipment, sutures, devices, and surgical stage. - The degree of novelty for a surgical technique/device should be mentioned (e.g. 'first in human' or 'first in this context'). - Medical devices should have manufacturer and model specifically mentioned. | 2,3,4 |
|  | **5h** | Operator Details   - Where applicable, include operator experience and position on the learning curve, any relevant training, and specialisation (e.g. ‘junior trainee with three years of surgical specialty training in Plastic Surgery and seven similar cases completed previously under direct supervision’). | 2,3,4 |
|  | **5i** | Quality Control   - What measures were taken to reduce inter- or intra-operator/operation variation, to ensure quality, and to maintain consistency between cases (e.g. independent observers, lymph node counts, standard surgical technique). - State any specific disparities between cases. | 2,3,4 |
|  | **5j** | Follow-Up   - When (e.g. how long after discharge, frequency, maximum follow-up length at the time of submission). - Where (e.g. home via video consultation, primary care, secondary care). - How (e.g. telephone consultation, clinical examination, blood tests, imaging). - Any specific long-term surveillance requirements (e.g. imaging surveillance of endovascular aneurysm repair or clinical exam/ultrasound of regional lymph nodes for skin cancer). - Any specific post-operative instructions (e.g. post-operative medications, targeted physiotherapy, psychological therapy). - State if any participants were lost to follow-up and why. | 2,3,4 |
| **Results** | **6a** | Participants   - Please state the number of patients involved, the patient characteristics (e.g. demographics, comorbidities, smoking status, and if applicable, tumour staging (e.g. TNM)). | 2,3,4 |
|  | **6b** | Deviation from the Initial Management Plan   - State if there were any changes in the planned intervention(s) (e.g. what was changed and why). - Please include a suitable schematic diagram if appropriate. | 2,3,4 |
|  | **6c** | Outcomes and Follow-Up   - Expected versus attained clinical outcome as assessed by the clinician. Reference literature used to inform expected outcomes. - When appropriate, include patient-reported measures (e.g. questionnaires including quality-of-life scales). - Describe and explain the percentage of patients lost to follow-up. | 2,3,4 |
|  | **6d** | Intervention Adherence and Compliance   - Where relevant, detail how well the patient adhered to and tolerated the advice provided (e.g. avoiding heavy lifting for abdominal surgery, or tolerance of chemotherapy and pharmacological agents). - Explain how adherence and tolerance were measured. | 2,3,4 |
|  | **6e** | Complications and Adverse Events   - Precautionary measures taken to prevent complications (e.g. antibiotic or venous thromboembolism prophylaxis). - All complications and adverse or unanticipated events should be described in detail and ideally categorised in accordance with the Clavien-Dindo Classification (e.g. blood loss, length of operative time, wound complications, re-exploration or revision surgery, impact on length of stay). - If relevant, was the complication reported to the relevant national agency or pharmaceutical company. - Specify the duration of time between completion of the intervention and discharge, and whether this was within the expected timeframe (if not, why not). - Where applicable, the 30-day post-operative and long-term morbidity/mortality may need to be specified. - State if there were no complications or adverse outcomes. | 2,3,4 |
| **Discussion** | **7a** | - Summarise the key results. | 4 |
|  | **7b** | Relevant Literature and Placing the Results in Context   - Include a discussion of the relevant literature and, if appropriate, similar published studies. - Describe the implications for clinical practice guidelines (e.g. NICE) and any relevant hypotheses generated. | 4,5,6 |
|  | **7c** | Strengths   - Describe the relevant strengths of the study. - Detail any multidisciplinary or cross-speciality relevance.   Weaknesses and Limitations   - Describe the relevant weaknesses or limitations of the study. - For novel techniques or devices, outline any contraindications and alternatives, potential risks and possible complications if applied to a larger population. | 4,5,6 |
|  | **7d** | Directions for Future Research   - State how the methodology and findings discussed can impact future research and clinical practice. Describe the questions that have arisen as a result of this study. - State the alternative study design(s) best suited to address these questions. | 4,5,6 |
| **Conclusions** | **8a** | Key Conclusions   - Outline the key conclusions from this study. | 6 |
|  | **8b** | Rationale   - Ensure that any of the conclusions made are supported by a strong rationale. | 6 |
|  | **8c** | Future Work   - Briefly discuss any questions arisen from this study and any differences in approach to patient diagnosis or management which the authors might adopt in future similar studies. | 6 |
| **Patient Perspective** | **9** | - Where appropriate, the patients should be given the opportunity to share their perspective on the intervention(s) they received (e.g. sharing quotes from a consented, anonymised interview, or questionnaire). | - |
| **Informed Consent** | **10** | - The authors must provide evidence of consent, where applicable, and if requested by the journal. - State the method of consent at the end of the article (e.g. verbal or written). - If not provided by the patients, explain why (e.g. death of patient and consent provided by next of kin). If the patients or family members were untraceable then document the tracing efforts undertaken. | 6 |
| **Additional Information** | **11a** | - State any conflicts of interest. | 7 |
|  | **11b** | - State any sources of funding. | 6 |
|  | **11c** | Other Relevant Disclosures   - Please state any author contributions, acknowledgments, and where required, institutional review board and ethical committee approval. - Disclose whether the case has been presented at a conference or regional meeting. | 6 |
| **Clinical Images and Videos** | **12** | - Where relevant and available, include clinical images to help demonstrate the cases pre-, peri-, and post-intervention (e.g. radiological, histopathological, patient photographs, intraoperative images). - Where relevant and available, include a link (e.g. Google Drive, YouTube) to the narrated operative video to highlight specific techniques or operative findings. - Ensure all media files are appropriately captioned and indicate points of interest to allow for easy interpretation. | 8,9,10,11 |
| **Referencing the Checklist** | **13** | - Include reference to the PROCESS 2020 publication by stating: 'This case series has been reported in line with the PROCESS Guideline' at the end of the methods section (and include citation in the references section). | 2 |
